# Occupational exposure to sharp instrument injuries in a federal hospital

**DOI:** 10.47626/1679-4435-2020-515

**Published:** 2021-03-03

**Authors:** Elinaldo Leite Quixabeiro, Élida Azevedo Hennington

**Affiliations:** 1 Divisão de Saúde do Trabalhador, Instituto Nacional de Câncer José Alencar Gomes da Silva, Rio de Janeiro, RJ, Brazil; 2 Escola Nacional de Saúde Pública Sergio Arouca, Rio de Janeiro, RJ, Brazil

**Keywords:** occupational accidents, health care providers, workers’ health surveillance, workers’ health, sharps injuries

## Abstract

**Introduction:**

Occupational exposure to potentially contaminated sharp instruments can result in the transmission of several pathogens and diseases. It is therefore necessary to investigate the factors that lead to these events and the interventions that can be used to address them.

**Objectives:**

To assess and describe the frequency of occupational exposure to sharps injury among employees of the Instituto Nacional de Câncer José Alencar Gomes da Silva in 2017 and 2018.

**Methods:**

This was a cross-sectional, exploratory and descriptive study based on the records of occupational accidents involving exposure to biological materials and the reports made to the Institute’s Occupational Health Division.

**Results:**

A total of 108 incidents were reported, 87 of which involved sharp instruments. Most accidents occurred in the surgical ward, and involved medical staff. The findings revealed low adherence to clinical and laboratory follow-up protocols. There were several errors in data entry and high rates of missing data for important characteristics of the accidents and victims, which prevented a more thorough description of these incidents.

**Conclusions:**

This study revealed several aspects of occupational accidents, victims and follow-up procedures, allowing for the discussion of strategies that could improve the reporting, prevention and management of these events.

## INTRODUCTION

Health care workers have a high risk of occupational exposure to sharps injuries in their daily professional practice. These risks are even higher in the hospital environment due to the high frequency with which invasive procedures are performed in this setting. In the context of health care work, sharps injuries are defined as events involving the accidental penetration of the skin by a sharp object. In the United States, the National Surveillance System for Health Care Workers (NaSH) reports that 80% of these injuries are caused by six types of object: disposable syringes, hypodermic needles, suture needles, winged-steel needles, intravenous catheter stylets and phlebotomy needles.^1^ Exposure to potentially contaminated body fluids as a result of these injuries can result in the transmission of over 20 different pathogens, with hepatitis B, C and the human immunodeficiency virus (HIV) being among the most common.^2^

The global population of health care workers, which is of approximately 35 million people,^2^ is thought to suffer nearly 3 million sharps injuries a year. In Brazil, though studies report a high rate of occupational exposure to sharps injuries,^3-8^ there are no real estimates of the number of workers affected by these incidents. Occupational accidents with exposure to biological material are usually reported to the Information System for Notifiable Diseases (SINAN), but the section of the report form where individuals are asked to describe the circumstances of the accident is often left blank.

After an occupational accident involving a sharp instrument, a report must be filed, and the worker involved must receive adequate medical assistance, including an analysis of the accident, preventive and prophylactic measures, as well as any additional procedures required by the institution.^9^ However, previous studies have found that over 50% of health care workers who experience this type of accident do not report its occurrence,^1^ preventing an accurate assessment of the situation and the adoption of preventive measures. According to the literature, several factors contribute to the underreporting of these incidents, including a low perception of contamination risk, unfamiliarity with reporting systems,^10^ the perception of the accident as irrelevant and unfamiliarity with the health care protocols for assisting victims of occupational accidents.^11^

The scientific literature on accidents with exposure to biological material (including sharps injuries) in Brazilian hospitals consists mostly of quantitative studies that attribute the accidents to the behavior of workers themselves. The focus on individual behavior rather than working conditions and occupational processes as the causes of occupational accidents denotes a casuistic approach that is rooted in the historical study of these issues. In Brazil and several other countries, the prevailing attitude of government institutions, employers and the press is that occupational accidents are caused by the workers themselves, due to negligence, distraction or dissatisfaction with work, thereby excluding organizational issues and increasingly precarious working conditions as potential causes of accidents.^12^

Silva^13^ notes that some aspects of work organization in hospitals may contribute to the occurrence of occupational accidents. These include the progressive intensification of work, the need to perform multiple simultaneous activities and the presence of frequent interruptions, in addition to other aspects of the collective management of current challenges. Factors related to sharp instruments themselves may also influence accident risk. A study conducted in an American hospital found that sharp instruments that require manipulation or disassembly after use were associated with a higher rate of accidents than syringes with disposable needles.^1^

Several initiatives can be implemented to address occupational exposure to potentially contaminated sharp instruments. Workers must be made aware of the risks to which they are exposed in their daily work environment, and of the need to care for their own safety and that of their colleagues. Health care services should promote workers’ health and focus on accident prevention, ensuring that all initiatives involve workers’ knowledge and participation. In light of these observations, the aim of this study was to assess and describe the occurrence of sharps injuries in employees of the Instituto Nacional de Câncer José Alencar Gomes da Silva (INCA) in 2017 and 2018, and discuss strategic interventions to address these accidents.

## METHODS

This was a cross-sectional, exploratory and descriptive study on occupational accidents involving exposure to sharp instruments recorded at the INCA between January 2017 and December 2018. The INCA specializes in cancer care, and comprises four separate sites (Cancer Hospital 1 [CH1], Cancer Hospital 2 [CH2], Cancer Hospital 3 [CH3] and Cancer Hospital 4 [CH4]) at different addresses in the city of Rio de Janeiro, which collectively employ approximately 3,600 workers.

Information on exposure incidents was collected in two stages. The first stage involved accessing a database of occupational accidents involving biological material in the institutional intranet. This database contains information on the workers, the accidents, and all relevant actions taken after the incidents. The records of these events were then retrieved from the Occupational Health Division (OHD), a service that provides assistance to health care workers. This method of data collection was chosen because gathering data from the OHD alone would exclude all accidents where victims did not report to this division to complete the recommended follow-up procedures.

Institutional protocols at the INCA recommend that all workers who have occupational accidents involving exposure to biological material be immediately evaluated by the on-call doctor at the First Aid Service (FAS) in the hospital where the incident occurred. During the assessment at the FAS, the doctor records the incident in the intranet database by filling out a Report Form for Accidents Involving Biological Material, in addition to providing the necessary assistance to the worker. After the initial medical consultation, the worker is referred to an occupational physician at the OHD.

The following data were collected for each incident: gender, age range, length of employment, occupation and sector of the worker; clinical site, date and time of incident, type of fluid, object, body part involved, type of exposure, circumstances, use of personal protective equipment (PPE), evolution of serological results and availability of OHD reports for the accident. These variables were selected in order to obtain a better understanding of the circumstances under which the incidents occurred. The data were stored in an electronic database and organized into tables, after which the incidents were analyzed and described. Only those involving full-time employees of the INCA were included in this study. Reports filed on behalf of outside contractors were excluded, since accidents involving these individuals are managed according to different protocols, where the INCA is only responsible for providing initial medical care. All subsequent follow-up assessments fall under the responsibility of the workers’ primary employer.

The present study was approved by the Research Ethics Committee of the Escola Nacional de Saúde Pública Sérgio Arouca/Fundação Oswaldo Cruz and the INCA, under protocol numbers 2.771.058 and 2.873.753, respectively.

## RESULTS

A total of 108 incidents involving biological materials were recorded in the database. Eighty-seven of these were caused by sharp instruments. The accidents and their characteristics are described in Table 1. Female workers were most affected, accounting for 57.5% of incidents. The distribution of accidents by age group revealed that most cases involved workers aged 20 to 30 (25.3%) or 31 to 40 years (28.7%). Accidents appear to decrease with advancing age. Most incidents involved medical staff (62.2%) and workers who had been in their current position for approximately 1 year. The sector with the highest number of incidents (40.2%) was the surgical department. When accidents were categorized by clinical site, results showed that 92% of incidents occurred in CH1.

**Table 1:** Distribution of sharps injuries by sex, age group, length of employment, occupation, department and site, Instituto Nacional de Câncer José Alencar Gomes da Silva, January 2017 to December 2018

	n	%
Gender		
Male	37	42.5
Female	50	57.5
Total	87	100.0
Age group		
20 to 30 years	22	25.3
31 to 40 years	25	28.7
41 to 50 years	11	12.7
51 to 60 years	9	10.3
> 60 years	4	4.6
Not reported	16	18.4
Total	87	100.0
Length of employment		
1 year	21	24.0
2 years	3	3.5
5 years	8	9.2
6 years	10	11.5
7 to 10 years	8	9.2
> 10 years	11	12.7
Not reported	26	29.9
Total	87	100.0
Occupation		
Nurse	13	14.9
Resident nurse	2	2.3
Nursing technician	17	19.5
Medical staff	31	35.7
Resident physician	23	26.5
Not reported	1	1.1
Total	87	100.0
Department		
Outpatient care	1	1.1
BMTC	1	1.1
Surgery	35	40.2
Adult ICC	3	3.5
Emergency care	5	5.8
Endoscopy	6	6.9
Inpatient care	16	18.4
Nuclear medicine	3	3.5
Chemotherapy	1	1.1
Radiology	3	3.5
Not reported	13	14.9
Total	87	100.0
Clinical site		
CH1	80	92.0
CH2	6	6.9
CH3	1	1.1
CH4	0	0.0
Total	87	100.0

Source: INCA Notification System for Accidents Involving Biological Material, 2018.

BMTC = Bone Marrow Transplantation Center; CH1, 2, 3 and 4 = Cancer Hospital 1, 2, 3 and 4; ICC = intensive care center.

As shown in Table 2, most incidents took place in the daytime (84%). Blood was cited as the contaminant in 69% of cases, and needles were involved in 21.8% of incidents. The most commonly injured body parts were the fingers (20.7%). The circumstances of the incident were only described in 11.5% of cases. Only 23% of workers reported to the OHD after their accidents.

**Table 2:** Distribution of sharps injuries by time of occurrence, contaminant, object involved, body part injured, circumstances and Occupational Health Division report, Instituto Nacional de Câncer José Alencar Gomes da Silva, January 2017 to December 2018

	n	%
Time of occurrence		
Day	73	84.0
Night	14	16.0
Total	87	100.0
Contaminant		
Bloody fluid	1	1.1
Pleural/peritoneal fluid	1	1.1
Blood	60	69.0
Tissue	1	1.1
Not reported	24	27.7
Total	87	100.0
Object		
Needle	19	21.8
Blood	11	12.7
Not reported	57	65.5
Total	87	100.0
Body part injured		
Finger	18	20.7
Left hand	1	1.1
Not reported	68	78.2
Total	87	100.0
Accident circumstances		
Disposal	3	3.5
Blood collection	2	2.3
Suturing	2	2.3
Blood glucose test	1	1.1
Intravenous catheter removal	2	2.3
Not reported	77	88.5
Total	87	100.0
OHD report		
Yes	20	23.0
No	67	77.0
Total	87	100.0

Source: Notification System for Accidents Involving Biological Material and INCA Occupational Health Division, 2018.

OHD = Occupational Health Division.

Serological test results (Table 3) showed that 92% of accident victims underwent serological testing on the day of the accident. However, only a single worker completed a second test 6 weeks after the accident, as recommended. The third serological test, to be completed 3 months after the accident, was only reported for two workers. Lastly, the fourth examination, which is to be scheduled 6 months after the accident, was only completed by one worker.

**Table 3:** Distribution of sharps injuries by serological test results, Instituto Nacional de Câncer José Alencar Gomes da Silva, January 2017 to December 2018

	n	%
Serological tests		
First test	80	92.0
Second test	1	1.1
Third test	2	2.3
Fourth test	1	1.1

Source: INCA Occupational Health Division, 2018.

## DISCUSSION

The present study showed that women were most affected by sharps injuries, with female workers accounting for 57.5% of cases. This may be explained by the fact that most workers in the health care sector and the institution examined are female. Similar results have been obtained in previous studies of Brazilian hospitals,^14^ a hospital in northern Thailand,^15^ and a university hospital in Serbia.^16^ The predominance of females in the health care sector has been described by Wermelinger et al.^17^ as a global phenomenon. In Brazil, the author noted that census data from the year 2000 showed that the vast majority of health care workers were female, with women accounting for nearly 70% of the workforce in this sector.

A significant number of incidents also affected resident physicians (26.5%) and workers who had been at the INCA for approximately 1 year (24%). These individuals may not have the necessary skills and training to perform their activities. This could be due to low-quality education, inexperience or poor working conditions. With regard to resident physicians, it is important to note that although these individuals are still in training, they tend to be on the front lines of patient care, and often perform high-complexity procedures. A study by Ottobelli et al.^18^ also observed a higher frequency of accidents in workers with less time and experience in their current positions. National and international studies have also shown that medical staff members account for a higher percentage of accidents involving sharp instruments.^11,19^

When the data were classified by hospital department, findings showed that the surgery ward had the highest incidence of accidents (40.2%), possibly due to the number of patients who go through surgery and the frequency with which the surgeons must handle sharp instruments under high-risk, high-pressure conditions. Operating rooms often have large pieces of technological equipment, leaving little room for workers to move around safely during procedures. Some types of surgery also require additional equipment, the use of devices that can weigh up to 20 kg, and the presence of specialized personnel. Many procedures also require the team to remain in the operating room for over 10 hours. These features are characteristic of the work in the surgical department, which is a cause of concern, since they also increase the risk of accidents. This poses a challenge for workers’ health initiatives and interventions targeting causal pathways and risk factors for work-related accidents and injury. This may be the reason why the present findings and those of previous studies point to surgical departments as the settings with the highest number of sharps injuries.^15,20^

The assessment of incidents per clinical site revealed that CH1 accounted for the highest proportion of incident reports (92%). This is indicative of underreporting in the other locations, as well as an unusually high prevalence of occupational accidents in CH1. Though this facility has the largest workforce and sees the largest number of patients, the level of activity at other units during the study period suggests that incidents there must have gone unreported. There is, as such, a need for a more detailed study of this issue in the other hospitals. Most accidents (84%) occurred in the daytime. This might be due to the faster pace of work during the day, and the increased number of procedures such as surgeries. These data corroborate the results of studies conducted in Portugal^21^ and Brazil.^22,23^ Blood was cited as the most common contaminant, and was involved in 69% of incidents, which is in line with previous findings in the literature.^14,20^

In the first stage of data collection for the present study, some variables were found to have high levels of missing data (object, body part injured, and accident circumstances), since the Report Form for Accidents Involving Biological Material, used to enter information into the intranet database, does not contain entry fields for additional information on the accident and the victim. The lack of information on these and other variables prevented a more thorough analysis of events, since few occupational accident victims reported to the OHD, which would have allowed for a more detailed assessment of the incidents.

The OHD is located near the CH1, in the central part of the city of Rio de Janeiro and far from the other clinical centers (CH2, CH3 and CH4), all of which are located in the northern part of the city. One reason why workers may not report to the OHD after their accidents to complete the clinical and laboratory follow-up may be the absence of such a department within the hospitals themselves and/or near their current jobs. This may be especially true for workers in the northern centers. This hypothesis is supported by our findings, which show that only 23% of workers reported their accidents to the OHD. These reports were used to collect additional data, which are relevant to the development of accident intervention programs.

### 1. Needlestick injuries.

The activities performed by health care workers, many of which are invasive procedures involving sharp instruments (including hollow-bore needles), are especially conducive to accidents. Current guidelines recommend that sharp instruments be replaced with devices that offer greater protection to workers whenever possible.

### 2. Fingers were the most frequently injured body parts, with finger injuries present in 20.7% of incidents.

This may be due to the nature of the work itself, which requires the use of hands and fingers to manipulate equipment and perform health-related procedures. Recommendations such as avoiding the use of fingers for support during procedures involving sharp instruments might help prevent accidents.

### 3. Most incidents occurred during the disposal of sharp objects, followed by blood collection.

With regards to disposal procedures, it is important that workers avoid placing sharp instruments in overfilled disposal boxes; pushing objects into the boxes during disposal; moving sharps boxes to make room for more material; and, especially, keeping the boxes far from the place where the residue is produced. As for blood collection, the most commonly used method still involves the use of needles and syringes to draw blood and transfer it to a collection tube, without placing the hands on any surface for support. This procedure is associated with increased risk to workers and other members of the medical team. The identification and monitoring of these behaviors is crucial for the development of accident prevention strategies.

### 4. Workers identified several causal factors and circumstances that increase the likelihood of accidents: fatigue and distraction, full disposal bins, lack of attention during disposal, and inadequate material for blood collection and transfer.

These factors and situations refer to work organization and working conditions. They might be especially frequent in precarious contexts, where workers are unaware of the risks intrinsic to their activities. Working conditions and type of occupational activity are strong determinants of worker health and a major cause of occupational accidents. Health care workers must continuously cope with the pain, suffering and death of patients. This situation, associated with the complexity of certain technical actions, the responsibility level and speed inherent in some decisions, work regimen (...), precarious bonds at work, the pressure and demands work entails and competitive relations in the work environment, represent a permanent aggression to these workers.^21^

### 5. All workers reported using PPE at the time of the accident.

The use of PPE is influenced by three factors, which include organizational issues.^23^ In order to improve adherence to PPE use, institutions must ensure access and availability of PPE that is compatible with the activities performed by workers and is sufficiently comfortable. Employers are also responsible for providing instructions and supervision of PPE use.

It is important to note that the complexity of accidents extends beyond the presence or absence of PPE use. Accident prevention also requires control and participative management strategies to improve the organization of work processes and working conditions.

### 6. Some workers reported previous training in their activities, but this was not always the case.

The development of institutional strategies that involve the provision of training before employees begin work is crucial for effective work performance and accident prevention. However, these strategies must reach all workers and be part of a continuous process, which adapts to changes in knowledge and identifies new sources of risk, in addition to focusing on specific themes that are relevant to the activities of each worker.

### 7. Some workers reported that neither they nor the on-call physicians who assessed them were familiar with the health care protocols for occupational accidents involving biological material at the INCA, or the procedures to be followed in these cases.

The present findings showed that, although there are protocols to manage biological exposure incidents at the INCA, these are not known to many of the workers, who are also unfamiliar with the procedures to be followed after an accident occurs. This calls attention to the need to promote continuous initiatives to disseminate information on these protocols and any updates, especially regarding the provision of immediate assistance to workers with occupational accidents.

It is important that all workers, especially those who provide assistance to individuals who experience accidental exposure to biological materials and potentially contaminated sharps, are trained in the INCA protocol, learning to fill out the forms (Table 1 provides clear evidence of incorrect data entry: ‘blood’ was often reported as the ‘object’ involved in the accident), follow the recommended steps and refer workers to the correct locations. Managers and continuing or in-service education departments must be involved in these initiatives.

## CONCLUSION

A total of 87 incidents involving sharp instruments were reported in the study period, with 21 of these involving biological fluids. The analysis of the total number of incidents and the near absence of reports from clinical sites CH2, CH3 and CH4, which employ over 3,000 workers, is indicative of underreporting.

The medical staff was most affected by these incidents. The discrepancy between this finding and those of previous studies, which have found nursing staff to report the highest number of accidents, may be attributable to underreporting. A significant number of workers did not report their accidents and/or complete their follow-up care at the OHD, as recommended by institutional protocols. This reaffirms the need for strategies to raise awareness of the protocol and the worker assistance procedures.

This study underscored the importance of improving accident reporting systems, since collecting quality data is an important step in the development of any intervention. There is a clear need to raise awareness among workers of the importance of reporting, preventing and managing sharps injuries, as well as developing permanent training and education programs to address these issues within the institution. In light of these findings, although sharps injuries are frequent and inherent to the professional practice of health care workers, the analysis of these occurrences, the development of prevention strategies and the medical follow-up of these instances still represent a challenge.
